# Cognitive Outcomes After Anterior Communicating Artery Aneurysm Repair

**DOI:** 10.1017/cjn.2018.16

**Published:** 2018-05-09

**Authors:** Farshad Nassiri, Adriana M. Workewych, Jetan H. Badhiwala, Michael D. Cusimano

**Affiliations:** Division of Neurosurgery, St. Michael’s Hospital, University of Toronto, Toronto, Ontario, Canada

**Keywords:** Aneurysms, Cerebrovascular surgery, Endovascular coiling, Cognitive impairment, Quality of Life

## Abstract

**Background** The long-term cognitive and quality-of-life (QoL) outcomes after treatment of ruptured anterior communicating artery (ACoA) aneurysms are unknown. **Methods** Potential participants were all consecutive patients with ruptured ACoA aneurysms who were treated at one institution from July 1992 to December 2008. All potential participants were asked to complete the Cognitive Failures Questionnaire (CFQ), Center for Epidemiology Studies-Depression (CES-D) questionnaire, Short Form 36 (SF-36) questionnaire, and Telephone Interview for Cognitive Status-Modified (TICS-M). Patient charts were retrospectively reviewed for baseline demographics and clinical status, intra-operative details, and post-operative course. Reporting of cognitive and QoL assessment results was stratified by treatment method (endovascular coil embolization and surgical clipping by pterional craniotomy or orbitocranial craniotomy). **Results** In total, 82 patients (18 treated with coiling, 27 by orbitocranial craniotomy, and 37 by pterional craniotomy) were included in this study. In total, 32 patients (9 treated by coiling, 11 by orbitocranial craniotomy, and 16 by pterional craniotomy) completed follow-up cognitive and QoL questionnaires. The mean follow-up for patients who completed the questionnaires was 8.64±3.81 years. The three groups did not differ in questionnaires assessing cognitive status (TICS-M *p*=0.114, CFQ *p*=0.111). Moreover, there were no observed differences in QoL or depression scores between the three groups. **Conclusions** At long-term follow-up, QoL, cognitive, and depression test scores of patients with ruptured ACoA aneurysms are similar across open surgery and coiling modalities. Our results emphasize the importance of considering long-term outcomes with validated daily measures of functioning when reporting on outcomes after treatment for ruptured intracranial aneurysms. Larger prospective studies are required to further explore the results.

## RÉSUMÉ

**Évolution des facultés cognitives après la réparation d’anévrismes de l’artère communicante antérieure.** ***Contexte:*** L’évolution à long terme des facultés cognitives et de la qualité de vie des patients ayant bénéficié d’un traitement à la suite d’une rupture d’anévrisme de l’artère communicantes antérieure demeure méconnue. ***Méthodes:*** Les participants potentiels à cette étude sont tous des patients s’étant présentés par ordre consécutif après avoir été victimes d’une rupture d’anévrisme de l’artère communicante antérieure. Fait à souligner, ils ont été traités dans un seul établissement de juillet 1992 à décembre 2008. On a demandé à tous ces participants de remplir une série de questionnaires : le *Cognitive Failures Questionnaire* (CFQ), celui du *Center for Epidemiology Studies-Depression* (CES-D), le SF 36 (de l’anglais *Short Form 36 Health Survey*) et le *Telephone Interview for Cognitive Status-Modified* (TICS-M). Les dossiers des patients ont ensuite fait l’objet d’une analyse rétrospective en tenant compte de leurs caractéristiques démographiques de base, de leur état clinique respectif, de considérations peropératoires et de leur évolution postopératoire. Le compte-rendu de l’évaluation de leurs facultés cognitives et de leur qualité de vie a été ensuite segmenté selon la méthode de traitement employée (l’embolisation par voie endovasculaire au moyen de spirales ou *coils* et le clippage chirurgical selon une crâniotomie ptérionale ou orbito-crânienne). ***Résultats:*** Au total, 82 patients (18 traités au moyen de spirales; 27, au moyen de la crâniotomie orbito-crânienne ; 37, au moyen de la crâniotomie ptérionale) ont été inclus dans cette étude. Sur ces 82 patients, 36 (soit 9 traités au moyen de spirales ; 11, au moyen de la crâniotomie orbito-crânienne; 16, au moyen de la crâniotomie ptérionale) ont rempli les questionnaires de suivi en lien avec leur qualité de vie et leurs facultés cognitives. La période moyenne de suivi pour les patients ayant rempli ces questionnaires a été de 8,64±3,81 ans. On n’a pas noté de grandes différences entre les trois groupes en ce qui regarde leur état cognitif (TICS-M *p*=0,114 ; CFQ *p*=0,111). Qui plus est, aucune différence n’a été observée en ce qui a trait à leur qualité de vie ou à leurs scores de dépression. ***Conclusions:*** Dans le cas d’un suivi à long terme de patients victimes d’une rupture d’anévrisme de l’artère communicante antérieure, on peut noter que tant leur qualité de vie, le maintien de leurs facultés cognitives que leurs scores à des test mesurant l’incidence de dépression sont semblables, et ce, peu importe qu’ils aient été traités en vertu d’une chirurgie ouverte ou de méthodes faisant appel à l’embolisation par voie endovasculaire au moyen de spirales. À la suite d’un traitement pour des ruptures d’anévrismes intrâcraniens, nos résultats mettent donc l’accent sur l’importance de prendre en considération l’évolution clinique à long terme de ces patients à l’aide d’indicateurs quotidiens et validés mesurant, au moment de leur récupération, leurs capacités à fonctionner. Des études prospectives plus importantes sont ainsi nécessaires pour effectuer une analyse plus approfondie de ces résultats.

## Introduction

Anterior communicating artery (ACoA) aneurysms are the most common intracranial aneurysms and the most common sources of aneurysmal subarachnoid hemorrhage (SAH).^1^ After the results of the International Subarachnoid Aneurysm Trial (ISAT) were published in 2002, endovascular coil embolization gained popularity for the treatment of ruptured intracranial aneurysms in most countries, where available.^2^ ^,^ ^3^ Considerable debate exists regarding the advantages and disadvantages of coiling versus clipping; however, this debate has mainly been based on short-term outcomes of procedural morbidity.^4^

Clinical outcomes after SAH are varied; however, neuropsychological deficits following SAH from rupture of ACoA aneurysms, such as memory, language, motor, and executive dysfunctions, are consistently reported.^5^ ^,^ ^6^ Despite the neuropsychological sequelae following aneurysmal SAH, there remains a relative paucity of literature on this topic compared with the more commonly assessed clinical and surgical outcomes after treatment.^7^ Moreover, whether treatment modality affects neuropsychological outcomes remains unclear. Previous work has shown memory and cognitive impairment in patients with ACoA aneurysms treated by surgical clipping.^8^ Furthermore, neuropsychological assessments of ISAT showed reduced tendencies for patients undergoing endovascular coiling to suffer cognitive impairment, which has been associated with depression and decreased quality of life (QoL).^9^ However, it is well established that the cognitive profile of these patients changes with time, and long-term cognitive and QoL outcomes across different treatment modalities have not been previously reported.^10^

Therefore, the goal of this study was to describe the long-term cognitive and QoL outcomes in patients with ruptured ACoA aneurysms treated by surgical clip occlusion of endovascular coil embolization.

## Material and Methods

This study was approved by the Research Ethics Board at St. Michael’s Hospital, Toronto, Ontario, Canada. We identified all consecutive patients at our center treated by open surgical clip occlusion or endovascular coil embolization for ruptured ACoA aneurysms from July 1992 to December 2008. In total, 82 potential participants were mailed a study information package outlining the details of the research study and containing self-reporting questionnaires to be filled out by the patient for long-term follow-up of the patient’s status. These questionnaires include the following: the Cognitive Failures Questionnaire (CFQ),^11^ the RAND Short Form 36 (SF-36),^12^ and the Centre for Epidemiological Studies-Depression (CES-D) questionnaire.^13^ The CFQ evaluates motor, perception, or memory abilities, whereas the SF-36 evaluates patient QoL over several measures. For the CFQ and SF-36, higher scores indicate poorer cognitive outcome and QoL, respectively. The CES-D evaluates depression, and scores of 16 or higher indicate individuals at a higher risk of depression.

Patients were subsequently contacted by telephone 2 weeks after the study information packages were distributed, and verbal consent to participate in further cognitive telephone based tests was obtained. For patients who agreed to participate, the Telephone Interview for Cognitive Status-Modified (TICS-M) questionnaire was administered in a standardized manner by one of our investigators as an objective assessment of global cognitive status.^14^ A score of 27 or lower is indicative of cognitive impairment.^15^

### Chart Review

The charts of all patients were reviewed. Demographic variables including age and sex, and baseline clinical factors including initial Glasgow Coma Scale (GCS) score and aneurysm size (largest dimension), were collected. The Hunt and Hess (HH) grading scale,^16^ the Fisher grade,^17^ and the World Federation of Neurological Surgeons (WFNS) grading system^18^ on admission were used to classify the severity of SAH. We recorded whether an external ventricular drain (EVD) was inserted before aneurysm treatment. For surgical procedures, we recorded whether an orbitocranial or standard pterional approach was used, the need for intra-operative EVD insertion, and whether the gyrus rectus was resected. We also recorded the occurrence of an intra-operative aneurysm rupture for all procedures. Moreover, we collected information on procedural complications (including hydrocephalus, meningitis, other non-cerebral infections including urinary tract infections, pneumonia, and bacteremia, stroke, hematoma as seen on post-procedural imaging, vasospasm and its treatment, hemiplegia, and post-procedural rupture), and the presence of an aneurysm remnant after repair, as determined by post-procedural imaging. Any repeated procedures for aneurysm treatment (re-clip or re-coil) were noted, as were any post-operative EVD re-insertions. We collected hospital discharge variables, comprising the hospital length of stay (LOS), intensive care unit (ICU) LOS, discharge GCS, and modified Ranking Scale (mRS).^19^ Modified Ranking Scale was determined retrospectively through comprehensive available chart data. We also document the follow-up duration, which was measured in years from the hospital admission date to the date of last contact with the patient.

### Statistical Analyses

Data were analyzed with IBM^®^ SPSS^®^ Statistics Version 24. We reported descriptive statistics as means and standard deviations, or median and interquartile range for continuous variables, or as frequencies and percentages for categorical variables. Glasgow Coma Scale is reported as a median value. Patients were stratified according to the type of treatment they received for their ruptured aneurysm (endovascular coiling, orbitocranial craniotomy and clipping, pterional craniotomy, and clipping). The Kruskal-Wallis test was used for our statistical analyses of continuous variables, and we similarly used Fisher’s exact test for categorical variables when comparing demographic variables, clinical, and questionnaire outcomes among the treatment groups, where appropriate, according to the normality of the data. Using a Bonferonni adjustment, *p*-values <0.017 (0.05/3) were considered statistically significant.

## Results

Of the 82 patients treated for ruptured ACoA aneurysms who were mailed questionnaire packages, 27 underwent orbitocranial craniotomy, 37 underwent pterional craniotomy, and 18 underwent endovascular coiling (see Table 1 of supplementary materials). Of these, 36 patients completed at least three of the four questionnaires (response rate of 43.9%). Of these patients, 9 underwent endovascular coiling, 11 underwent orbitocranial craniotomy and clipping, and 16 underwent pterional craniotomy and clipping. The mean follow-up period of the 36 patients who completed questionnaires was 8.64±3.81 years (range 1.43-15.95 years).

### Demographics and Clinical Outcomes

Table 1 presents the patient demographics and baseline clinical factors and outcomes comparing the patients who did versus those who did not complete questionnaires. The two groups were similar in baseline characteristics. The distribution of HH Stroke Scale, Fisher, and WFNS scores was similar among those who did versus those who did not complete questionnaires (*p*=0.716, *p*=0.578, and *p*=0.853, respectively).

**Table 1:** Baseline demographics and clinical factors and outomes for patients with completed questionnaires compared with patients without questionnaire data (*n*=82)

	Completed questionnaires (*n*=36)	No questionnaires (*n*=46)	*p* value
Demographic and pre-procedural variables			
Sex (male)	18 (50%)	21 (46%)	0.696
Age (at treatment)	55.53±11.22	54.76±14.33	0.792
Admit GCS** (median)	15 (IQR 14-15) (range 4-15)	15 (IQR 14-15) (range 3-15)	0.661
HH Stroke Scale			0.716
1	14 (39%)	19 (41%)	
2	11 (30%)	15 (33%)	
3	5 (14%)	9 (19.5%)	
4	4 (11%)	3 (6.5%)	
5	1 (3%)	0 (0%)	
Missing	1 (3%)	0 (0%)	
Fisher Grade			0.578
1	7 (19%)	1 (2%)	
2	5 (14%)	3 (6.5%)	
3	10 (28%)	23 (50%)	
4	14 (39%)	10 (22%)	
Missing	0 (%)	9 (19.5%)	
WFNS Grading Scale			0.578
I	21 (58%)	30 (65%)	
II	9 (25%)	9 (20%)	
III	0 (0%)	0 (0%)	
IV	3 (8%)	4 (9%)	
V	2 (6%)	2 (4%)	
Missing	1 (3%)	1 (3%)	
Aneurysm size*** (mm)	6.67±3.06	6.44±3.30	0.771
Pre-operative EVD	5 (14%)	4 (9%)	0.496
Intra- and post-operative variables			
Initial post-procedure GCS****	14 (IQR 11-15)	14 (IQR 10-15)	0.832
Intra-operative rupture	12 (33%)	11 (24%)	0.346
Gyrus rectus resection	5 (14%)	3 (7%)	0.290
Intra-operative EVD	10 (28%)	17 (37%)	0.380
Repeat procedure for aneurysm security	10 (28%)	4 (9%)	0.023
Infection	12 (33%)	16 (35%)	0.891
Hemiplegia	3 (8%)	3 (7%)	1.00
Stroke	4 (11%)	1 (2%)	0.163
Hematoma	3 (8%)	7 (15%)	0.501
Vasospasm	11 (31%)	24 (52%)	0.072
Residual aneurysm	10 (28%)	15 (33%)	0.637
Post-operative rebleed	0 (0%)	2 (4%)	0.501
Meningitis	1 (3%)	1 (2%)	1.00
Post-operative EVD insertion	17 (47%)	14 (30%)	0.120
Hydrocephalus	9 (25%)	15 (33%)	0.452
Discharge mRS*****			0.552
0-2	24 (67%)	31 (67%)	
3-6	10 (28%)	15 (33%)	
Missing	2 (6%)	0 (0%)	
Discharge GCS******	15 (IQR 15-15)	15 (IQR 13-15)	0.018
Hospital LOS******* (days)	21.68±14.79	30.13±25.85	0.154
ICU LOS******** (days)	11.50±9.65	14.96±12.71	0.234

EVD=External ventricular drain; GCS=Glasgow Coma Scale; HH=Hunt and Hess; LOS=length of stay; NA=not available; WFNS=World Federation of Neurological Societies. Data are mean±STD, median (range), or frequency (%). * indicates statistically significant values.

** Comparison contains: questionnaires completed *n*=34, no questionnaires *n*=46.

*** Comparison contains: questionnaires completed *n*=34, no questionnaires *n*=36.

**** Comparison contains: questionnaires completed *n*=26, no questionnaires *n*=46.

***** Comparison contains: questionnaires completed *n*=34, no questionnaires *n*=46.

****** Comparison contains: questionnaires completed *n*=25, no questionnaires *n*=44.

******* Comparison contains: questionnaires completed *n*=28, no questionnaires *n*=45.

******** Comparison contains: questionnaires completed *n*=26, no questionnaires *n*=45.

For the patients who completed questionnaires, the baseline clinical characteristics, clinical outcomes, and questionnaire outcomes were stratified according to treatment modality (see Tables 2-4, respectively). The demographics and baseline clinical characteristics did not differ significantly between patients treated by orbitocranial craniotomy and clipping, pterional craniotomy and clipping, and endovascular coiling (Table 2). Patients treated with pterional craniotomy and clipping had higher rates of intra-operative rupture when compared with patients treated by orbitocranial craniotomy and clipping and endovascular coiling (56% vs. 18% and 11%, respectively, *p*=0.045). Similarly, those treated with pterional craniotomy also had higher rates of gyrus rectus resection at the time of surgery when compared with orbitocranial craniotomy patients (31% vs. 0%, *p*=0.030). Otherwise, the clinical outcomes of the three groups were similar with respect to intra-operative use of EVD, post-procedural GCS, repeat procedure for aneurysm security, infection, hemiplegia, stroke, post-operative hematoma, vasospasm, residual aneurysm, post-operative aneurysm rebleed, meningitis, post-operative EVD insertion, hydrocephalus, discharge mRS, discharge GCS, hospital LOS, and ICU LOS (Table 3).

**Table 2:** Baseline demographics of patients with completed questionnaires (*n*=36)

Demographic and pre-procedural variables	Endovascular coiling (*n*=9)	Orbitocranial craniotomy (*n*=11)	Pterional craniotomy (*n*=16)	*p* value
Sex (male)	5 (56%)	7 (64%)	6 (38%)	0.381
Age (at treatment)	50.89±11.93	56.45±12.19	57.50±10.05	0.359
Age (at questionnaire completion)	57.30±11.64	63.62±12.85	64.86±10.60	0.289
Admit GCS** (median)	14.5 (IQR 13-15) (range 9-15)	15 (IQR 14-15) (range 4-15)	15 (IQR 14-15) (range 7-15)	0.640
HH Stroke Scale				0.656
1	4 (45%)	3 (27%)	7 (44%)	
2	2 (22%)	5 (46%)	4 (25%)	
3	3 (33%)	0 (0%)	2 (12.5%)	
4	0 (0%)	2 (18%)	2 (12.5%)	
5	0 (0%)	1 (9%)	0 (%)	
Missing	0 (0%)	0 (0%)	1 (6%)	
Fisher Grade				0.574
1	2 (22%)	3 (27%)	2 (12.5%)	
2	0 (0%)	1 (9%)	4 (25%)	
3	2 (22%)	4 (36%)	4 (25%)	
4	5 (56%)	3 (27%)	6 (37.5%)	
WFNS Grading Scale				0.328
I	4 (44.5%)	7 (64%)	10 (63%)	
II	4 (44.5%)	2 (18%)	3 (18%)	
III	0 (0%)	0 (0%)	0 (0%)	
IV	1 (11%)	0 (0%)	2 (13%)	
V	0 (0%)	2 (18%)	0 (0%)	
Missing	0 (0%)	0 (0%)	1 (6%)	
Aneurysm size*** (mm)	6.49±2.67	6.25±2.99	7.10±3.48	0.784
Pre-operative EVD	3 (33%)	0 (0%)	2 (13%)	0.099

EVD=external ventricular drain; GCS=Glasgow Coma Scale; HH=Hunt and Hess; NA=not available; WFNS=World Federation of Neurological Societies. Data are mean±STD, median (range), or frequency (%). * indicates statistically significant values.

** Comparison contains: coiling *n*=8, orbitocranial craniotomy *n*=11, pterional craniotomy *n*=15.

*** Comparison contains: coiling *n*=9, orbitocranial craniotomy *n*=11, pterional craniotomy *n*=14.

**Table 3:** Clinical outcomes for patients with comleted questionnaires (*n*=36)

Intra- and post-operative variables	Endovascular coiling (*n*=9)	Orbitocranial craniotomy (*n*=11)	Pterional craniotomy (*n*=16)	*p* value
Initial post-procedure GCS**	14.00 (IQR 8-15) (range 3-15)	13.50 (IQR 11-15) (range 8-15)	14.00 (IQR 13-15) (range 9-15)	0.492
Intra-operative rupture	1 (11%)	2 (18%)	9 (56%)	0.045
Gyrus rectus resection	0 (0%)	0 (0%)	5 (31%)	0.030
Intra-operative EVD	1 (11%)	4 (36%)	5 (31%)	0.511
Repeat procedure for aneurysm security	4 (44%)	2 (18%)	4 (25%)	0.404
Infection	2 (22%)	3 (27%)	7 (44%)	0.491
Hemiplegia	1 (11%)	1 (9%)	1 (6%)	1.00
Stroke	1 (11%)	1 (9%)	2 (13%)	1.00
Hematoma	1 (11%)	1 (9%)	1 (6%)	1.00
Vasospasm	1 (11%)	5 (46%)	5 (31%)	0.272
Residual aneurysm	2 (22%)	2 (18%)	6 (38%)	1.00
Post-operative rebleed	0 (0%)	0 (0%)	0 (0%)	–
Meningitis	0 (0%)	0 (0%)	1 (6%)	1.00
Post-operative EVD insertion	4 (44%)	5 (46%)	8 (50%)	1.00
Hydrocephalus	2 (22%)	3 (27%)	4 (25%)	1.00
Discharge mRS				0.121
0-2	7 (78%)	9 (82%)	8 (50%)	
3-6	2 (22%)	1 (9%)	7 (44%)	
Missing	0 (0%)	1 (9%)	1 (6%)	
Discharge GCS*** (median)	15 (IQR 15-15) (range 14-15)	14 (IQR 13-15) (range 13-15)	15 (IQR 15-15) (range 13-15)	0.044
Hospital LOS**** (days)	16.50±11.96	21.17±9.06	24.86±17.84	0.458
ICU LOS***** (days)	9.29±7.02	12.00±5.51	12.46±12.36	0.788
Follow-up duration (years)	6.41±2.06	7.16±2.64	7.36±3.44	0.734

EVD=external ventricular drain; GCS=Glasgow Coma Scale; GOSE=Glasgow Outcome Score-Extended; LOS=length of stay; mRS=modified Ranking Score; NA=not available. Data are mean±STD, median [range], or frequency (%). * indicates statistically significant values.

** Comparison contains: coiling *n*=7, orbitocranial craniotomy *n*=6, pterional craniotomy *n*=13.

*** Comparison contains: coiling *n*=7, orbitocranial craniotomy *n*=6, pterional craniotomy *n*=13.

**** Comparison contains: coiling *n*=8, orbitocranial craniotomy *n*=6, pterional craniotomy *n*=14.

***** Comparison contains: coiling *n*=7, orbitocranial craniotomy *n*=6, pterional craniotomy *n*=13.

**Table 4:** Questionnaire outcomes

Questionnaire	Endovascular coiling (*n*=9)	Orbitocranial craniotomy (*n*=11)	Pterional craniotomy (*n*=16)	*p* value
CES-D	17.44±8.23	8.45±6.92	12.88±10.78	0.107
CFQ	33.33±17.32	19.73±13.25	28.38±13.32	0.111
SF-36	64.54±31.49	69.17±17.86	57.62±20.80	0.436
Physical functioning	66.11±37.98	69.55±24.85	52.19±31.83	0.331
Energy and fatigue	55.00±29.47	50.60±19.72	47.81±19.32	0.744
Emotional well-being	65.78±27.87	76.36±14.91	71.50±22.81	0.574
Social functioning	69.44±30.04	78.40±25.06	77.34±27.08	0.712
Pain	68.88±42.50	76.14±31.39	51.72±34.09	0.126
General health	54.16±35.22	70.45±20.67	50.60±27.48	0.135
Role limitations owing to physical health	69.44±46.40	63.64±37.69	43.75±46.99	0.322
Role limitations owing to emotional problems	66.67±50.00	69.70±45.84	68.75±44.67	1.00
Health change	35.56±48.76	37.72±45.57	42.50±40.99	0.850
TICS*	34.17±2.99	29.33±5.72	29.54±4.59	0.114

CES-D=Center for Epidemiological Studies-Depression; CFQ=Cognitive Failures Questionnaire; SF-36=RAND Short Form 36; TICS=Telephone Interview for Cognitive Status. Data are mean±STD.

* Comparisons contains: coiling *n*=6, orbitocranial craniotomy *n*=6, pterional craniotomy *n*=13.

### Questionnaire Outcomes

Table 4 presents the results from the questionnaires administered to patients. There were no differences in any questionnaire outcomes among the three groups (*p*=0.107 for CES-D, *p*=0.111 for CFQ, *p*=0.436 for SF-36, *p*=0.114 for TIC-M).

There was a trend toward higher scores on the TICS-M in patients treated with endovascular coil embolization compared with surgery; however, this comparison did not reach statistical significance. TICS-M scores ≤27 have previously been used as a threshold to suggest cognitive impairment.^15^ There were 0, 2, and 4 patients treated by endovascular coiling, orbitocranial clipping, and pterional clipping, respectively, who had TICS-M scores ≤27.

Although not statistically significant, we also observed a trend toward higher scores on the CFQ and CES-D questionnaires in patients treated by endovascular coiling compared with orbitocranial craniotomy. There were 4, 3, and 5 patients treated by coiling, orbitocranial craniotomy, and pterional craniotomy, respectively, who had CES-D scores ≥16 (*p*=0·617), which was the previously indicated threshold suggestive of possible depression.

## Discussion

Surgically, aneurysms of the ACoA complex may be approached via standard pterional craniotomy, or with a modified orbitocranial craniotomy. By using an orbital osteotomy, the orbitocranial approach offers improved exposure of anterior structures, larger volume of access for improved surgeon maneuverability, less necessary brain retraction, and shorter working distances than the standard pterional craniotomy.^20^ Although the orbitocranial craniotomy allows for the technical advantages listed above, it is unclear whether these translate into long-term neuropsychological improvements for patients compared with standard pterional craniotomy.

In our study, we assessed cognitive functioning using the CFQ and TICS-M questionnaires, both of which are validated tools that have been previously used in patients with aneurysmal SAH.^9^ ^,^ ^21^ The TICS-M provides an objective measure of global cognitive functioning, whereas the CFQ provides subjective patient-reported accounts of failures in perception, memory, and motor function in everyday life. Our results demonstrated that the two surgical approaches performed similarly on the CFQ and TICS-M. Moreover, none of the patients in the endovascular-treated group had TICS-M scores lower than the previously established cutoff for cognitive impairment (≤27), whereas 2 and 4 patients in the orbitocranial and pterional craniotomy groups, respectively, had scores below this threshold. Higher intra-operative rates of rupture in patients treated with surgery and the less invasive nature of endovascular therapy may potentially be contributing to these observed differences. Open surgical clipping may necessitate brain retraction or parenchymal manipulation that may result in local tissue trauma that is not otherwise evident with coiling procedures.^22^ In addition, intra-operative aneurysmal rupture or compromise of parent or perforating arteries with any treatment modality is important in the development of neurological symptoms referred to as “ACoA Syndrome,”^23^ which encompasses memory deficits, personality changes, and confabulation. In our series, the rates of intra-operative rupture were higher with pterional craniotomy and clipping compared with either orbitocranial craniotomy or endovascular coiling.

Self-reported daily cognitive failures in patients treated with coiling were more frequent compared with those treated with orbitocranial craniotomy, although this comparison did not reach significance. Our study was not designed to further evaluate the reason for these discrepant results, and future prospective trials are indicated in order to explore these differences. It is possible that greater field of view and maneuverability offered by the orbitocranial approach allows for preservation of brain tissue that may be important in self-perception, or insight for cognitive functioning that may not be well preserved with standard pterional craniotomy. In our study, 29% of patients treated by pterional craniotomy and clipping and none of the patients treated by orbitocranial craniotomy and clipping had partial or complete gyrus rectus resection. As the gyrus rectus, as part of the medial prefrontal cortex, may play a role in higher cognitive functioning, it is possible that this difference in technique at least partly explains the differences in self-reported outcomes.^24^ However, the reason why endovascular-treated patients report higher subjective failures of cognition in everyday life remains to be answered and should be evaluated with future prospective trials.

Taken together, the data on our cognitive outcomes suggest that although local tissue damage from parenchymal manipulation with open surgery may alter outcomes to a degree, it is likely that the brain damage associated with the primary subarachnoid hemorrhage and complications related to SAH is the overriding determinant of outcomes, and that these subtle differences do not translate into significant effects noted by patients in everyday life. Overall, the results of our study support the previous literature that suggests that patients with ruptured ACoA aneurysms do experience impaired cognitive functioning, such as decision-making capabilities and memory.^25^ ^,^ ^26^

In our study, there were no statistically significant differences in results of SF-36 or CES-D scores in patients treated with coiling compared with surgery. There have been few studies assessing the long-term QoL outcomes in patients with ruptured aneurysms, and whether these outcomes are affected by treatment modality. Von Vogelsang et al^27^ reported that patients with ruptured intracranial aneurysms who were followed up for at least 10 years continued to show more problems in mobility, self-care, usual activities, and depression and anxiety compared with the general population. Moreover, similar to our study, Proust et al^28^ prospectively assessed QoL and cognition in patients with ACoA aneurysms using an extensive battery of tests and found no differences in mRS and QoL scores between treatment groups at 14 months post treatment. Previous studies report variable outcomes when assessing mood and depression in patients with ruptured aneurysms. Preiss et al^29^ compared patients treated for ruptured cerebral aneurysms via microsurgical clipping and coiling and found no differences in neuropsychological cognitive tests or mood tests at 1 year after treatment, whereas Katati et al^30^ assessed QoL outcomes in patients with ruptured cerebral aneurysms, with 26 ruptured ACoA patients, and found that nearly half of the patients suffered from anxiety or depression at 4 months after treatment. Although in this study there were no significant differences in QoL and depression scores, there was a trend to higher depressive tendencies in endovascular-treated patients compared with surgically treated patients, which interestingly parallels the higher self-reported daily cognitive failures in this group. Our study was not powered to explore this correlation in detail, and future prospective trials should be undertaken in order to determine whether this relationship is sustained and is causal.

The findings of this study should be interpreted in light of its limitations. First, clinical information was collected retrospectively and therefore was subjected to the limitations of the information available to the investigators. Some of our patients had unavailable clinical data, and this was noted in our results and dealt with appropriately for statistical comparisons. Moreover, not all patients completed every questionnaire that was administered, and several more patients in the coiling group completed the questionnaire than in the clipping group; this may represent an inherent reporting bias. However, comparison of baseline and clinical outcomes of patients who did versus those who did not complete questionnaires in this consecutive series of patients does not show any significant differences between the two groups. Our overall response rate to the questionnaires was 44%, and thus our results may be subject to selection bias.

The findings of this study should be interpreted in light of its limitations. First, clinical information was collected retrospectively and therefore was subjected to the limitations of the information available to the investigators (e.g., pre-SAH cognitive status was unable to be determined) and selection bias (patients with a poor prognosis who die in hospital following aneurysm rupture would not be able to be followed up in the long-term). Overall, our study was limited by its small sample size, which precluded meaningful age-matched comparisons for questionnaire data. However, it is noteworthy, given the effect size for questionnaire outcomes, that had the sample size been bigger the difference in questionnaire outcomes could have been meaningfully different between groups. As seen in Table 5, the number of patients in our analysis is similar to or larger than other studies that have assessed cognitive outcomes assessments in this population with much longer follow-up durations.^5^ ^,^ ^6^ ^,^ ^8^ ^,^ ^23^ ^,^ ^25^ ^,^ ^28^ ^,^ ^31^ It is worthwhile to note that mRS data were not documented prospectively, as this scale is not routinely used clinically at our institution. We therefore determined mRS retrospectively, and these values should be interpreted in light of this. Last, eligible patients were treated over a 16-year period (1992-2008), during which there may have been considerable advancements in procedural and intensive care management. However, in our analysis, patients in all three groups did not differ substantially in procedural complications or clinical outcomes that could be explained by advances in care for patients.

**Table 5:** Comparison of follow-up duration across studies testing cognition in ruptured ACoA patients

Author	Number of patients with ruptured anterior communicating artery aneurysms	Follow-up duration
Nassiri et al	36	103.7 months
Ravnik et al^6^	10	41 months
Escartin et al^25^	40	32.5-36.6 months
O’Connor et al^31^	14	24 months
Proust et al^28^	50	14 months
Haug et al^5^	24	12 months
Papagno et al^8^	27	0.4-12.6 months
DeLuca et al^23^	11	1-4 months

## Conclusion

This study is the first to compare the very long-term QoL, depression, and cognitive outcomes of patients treated for ruptured ACoA aneurysms by endovascular coil embolization, orbitocranial craniotomy and clip occlusion, or pterional craniotomy and clip occlusion. We showed that, regardless of treatment modality, self-reported QoL, depressive, and cognitive tests were similar in all groups. Our exploratory study results emphasize the importance of conducting future studies that evaluate long-term outcomes with validated daily measures of functioning in deciding on a management approach for ruptured aneurysms. Such long-term prospective trials are needed to better disentangle the interaction of depression, cognitive test scores, and practical real-world measures of daily cognitive functioning in patients with ruptured ACoA aneurysms.

## Supplementary material

For supplementary material accompanying this paper visit http://dx.doi.org/10.1017/cjn.2018.16. click here to view supplementary material
