# Supplementary material for: Cognitive Outcomes After Anterior Communicating Artery Aneurysm Repair
Source: Can J Neurol Sci. 2018 May 9;45(4):415–23. doi: 10.1017/cjn.2018.16 (PMC6088546; doi:10.1017/cjn.2018.16)
Supplement: Supplementary file 1 [file S0317167118000161sup001.docx]

**SUPPLEMENTAL TABLE 1. BASELINE DEMOGRAPHICS AND CLINICAL FACTORS AND OUTCOMES FOR ALL PATIENTS WHO WERE MAILED QUESTIONNAIRES STRATIFIED ACCORDING TO TREATMENT STRATEGY (n = 82)**

| Demographic and pre-procedural variables | | | Endovascular coiling (n=18) | Orbitocranial craniotomy  (n=27) | Pterional craniotomy  (n=37) | *P* value |
| --- | --- | --- | --- | --- | --- | --- |
| Sex (male) | | | 9 (50%) | 17 (63%) | 13 (35%) | 0.086 |
| Age (at treatment) | | | 51.22 ± 10.55 | 56.52 ± 12.34 | 57.35±10.88 | 0.160 |
| Admit GCS£ | | | 15 [IQR 14-15]  [RANGE 6-15] | 15 [IQR 14-15]  [RANGE 3-15] | 15 [IQR 14-15]  [RANGE 7-15] | 0.171 |
| HH Stroke Scale |  | |  |  |  | 0.715 |
|  | **1** | | 10 (56%) | 10 (37%) | 13 (35%) |  |
|  | **2** | | 3 (17%) | 9 (33%) | 13 (35%) |  |
|  | **3** | | 3 (17%) | 4 (15%) | 7 (19%) |  |
|  | **4** | | 1 (6%) | 3 (11%) | 3 (8%) |  |
|  | **5** | | 0 (3%) | 1 (4%) | 0 (0%) |  |
|  | **Missing** | | 1 (6%) | 0 (0%) | 1 (3%) |  |
| Fisher Grade | | |  |  |  | 0.480 |
|  | **1** | | 2 (11%) | 3 (11%) | 3 (8%) |  |
|  | **2** | | 1 (6%) | 3 (11%) | 4 (11%) |  |
|  | **3** | | 6 (33%) | 14 (52%) | 13 (35%) |  |
|  | **4** | | 6 (33%) | 7 (26%) | 11 (30%) |  |
|  | **Missing** | | 3 (17%) | 0 (0%) | 6 (16%) |  |
| WFNS Grading Scale | | |  |  |  | 0.241 |
|  | | **I** | 11 (61%) | 16 (59%) | 24 (65%) |  |
|  |  | **II** | 5 (28%) | 6 (22%) | 6 (16%) |  |
|  |  | **III** | 0 (0%) | 0 (0%) | 1 (3%) |  |
|  |  | **IV** | 2 (11%) | 1 (4%) | 4 (11%) |  |
|  |  | **V** | 0 (0%) | 4 (15%) | 0 (0%) |  |
|  |  | **Missing** | 0 (0%) | 0 (0%) | 2 (5%) |  |
| Aneurysm size¥ (mm) | | | 5.67 ± 2.44 | 7.36 ± 3.21 | 5.67 ± 2.44 | 0.214 |
| Pre-operative EVD | | | 3 (17%) | 3 (11%) | 3 (8%) | 0.534 |
| Intra- and post-operative variables | | | Endovascular coiling (n=18) | Orbitocranial craniotomy  (n=27) | Pterional craniotomy  (n=37) | ***P* value** |
| Initial post-procedure GCS& | | | 15 [IQR 9.5-15]  [RANGE 3-15] | 13.5 [IQR 11-15]  [RANGE 8-15] | 14 [IQR 11-15]  [RANGE 7-15] | 0.055 |
| Intra-operative rupture | | | 3 (17%) | 7 (26%) | 13 (35%) | 0.353 |
| Gyrus rectus resection | | | 0 (0%) | 0 (0%) | 8 (22%) | 0.290 |
| Intra-operative EVD | | | 1 (6%) | 11 (41%) | 15 (41%) | 0.020 |
| Repeat procedure for aneurysm security | | | 5 (28%) | 3 (11%) | 6 (16%) | 0.389 |
| Infection | | | 4 (22%) | 9 (33%) | 15 (41%) | 0.403 |
| Hemiplegia | | | 1 (6%) | 1 (4%) | 1 (6%) | 0.658 |
| Stroke | | | 1 (6%) | 1 (4%) | 3 (8%) | 0.846 |
| Hematoma | | | 2 (11%) | 1 (4%) | 7 (19%) | 0.232 |
| Vasospasm | | | 4 (22%) | 12 (44%) | 19 (51%) | 0.119 |
| Residual aneurysm | | | 6 (33%) | 7 (26%) | 12 (32%) | 0.819 |
| Post-operative rebleed | | | 1 (6%) | 0 (0%) | 1 (3%) | 0.699 |
| Meningitis | | | 0 (0%) | 1 (4%) | 1 (3%) | 1.00 |
| Post-operative EVD insertion | | | 5 (28%) | 11 (41%) | 15 (41%) | 0.611 |
| Hydrocephalus | | | 3 (17%) | 8 (30%) | 13 (35%) | 0.368 |
| Discharge mRS^ | | |  |  |  | 0.468 |
| 0-2  3-6  Missing | | | 15 (83%) | 18 (66%) | 22 (59%) |  |
|  |  |  | 3 (17%) | 8 (30%) | 14 (38%) |  |
|  |  |  | 0 (0%) | 1 (4%) | 1 (3%) |  |
|  |  |  |  |  |  |  |
| Discharge GCS! | | | 15 [IQR 15-15]  [RANGE 12-15] | 14 [IQR 13-15]  [RANGE 3-15] | 15 [IQR 13.5-15]  [RANGE 8-15] | 0.025 |
| Hospital LOS$ (days) | | | 15.5 ± 9.43 | 31.04 ± 28.46 | 29.49 ± 21.35 | 0.020 |
| ICU LOS¢ (days) | | | 9.75 ± 6.20 | 15.50 ± 14.94 | 14.39 ± 11.22 | 0.298 |

Data are mean ± STD, median [range], or frequency (%)
Asterisk (*) indicates statistically significant values
£ comparison contains: Endovascular coiling n=17, Orbitocranial craniotomy n=27, Pterional craniotomy n=36
¥ comparison contains: Endovascular coiling n=15, Orbitocranial craniotomy n=26, Pterional craniotomy n=29

**&** comparison contains: Endovascular coiling n=17, Orbitocranial craniotomy n=22, Pterional craniotomy n=34

**^** comparison contains: Endovascular coiling n=18, Orbitocranial craniotomy n=26, Pterional craniotomy n=36

**!** comparison contains: Endovascular coiling n=16, Orbitocranial craniotomy n=21, Pterional craniotomy n=32

**$** comparison contains: Endovascular coiling n=16, Orbitocranial craniotomy n=22, Pterional craniotomy n=35

**¢** comparison contains: Endovascular coiling n=16, Orbitocranial craniotomy n=22, Pterional craniotomy n=33

Abbreviations: EVD = External ventricular drain, GCS = Glasgow Coma Scale, HH = Hunt and Hess, NA = not available, WFNS = World Federation of Neurological Societies
